# Prevalence of high-risk for advanced liver fibrosis using non-invasive scores among type 2 diabetes mellitus patients in the United Arab Emirates: a retrospective cross-sectional study

**DOI:** 10.3389/fendo.2026.1719626

**Published:** 2026-02-17

**Authors:** Najma Yaqoob, Shaikha Salah Alhaj, Ayah Maher Al Obid, Amna Alketbi, Meera Alsuwaidi, Alia Ali Galadari, Amna Ahmad, Heitham Ajlouni, Dima Kamal Abdelmannan

**Affiliations:** 1 Graduate Medical Education, Dubai Health, Dubai, United Arab Emirates; 2 Consultant Endocrinologist, Dubai Health, Dubai, United Arab Emirates

**Keywords:** liver fibrosis, liver fibrosis risk, prevalence, type 2 diabetes mellitus, United Arab Emirates

## Abstract

**Background:**

Advanced liver fibrosis is a major determinant of liver-related morbidity and mortality and occurs more frequently in individuals with type 2 diabetes mellitus (T2DM). While metabolic dysfunction–associated steatotic liver disease (MASLD) is highly prevalent among patients with T2DM, data on the risk of advanced liver fibrosis in this population in the United Arab Emirates (UAE) remain limited. Non-invasive fibrosis scores offer a pragmatic approach for risk stratification in routine clinical practice.

**Aims:**

To determine the prevalence of high risk for advanced liver fibrosis and its clinical associations among patients with T2DM in the UAE using validated non-invasive fibrosis scores.

**Methodology:**

A retrospective cross-sectional study was conducted between October to December 2023, enrolling T2DM patients who attended at the Diabetic Care Centre in Dubai, UAE. Data was extracted from the Dubai Health electronic medical records, Epic (Salama). Liver fibrosis risk was assessed using the fibrosis-4 (FIB-4) score and Nonalcoholic Fatty Liver Disease (NAFLD) fibrosis score, with chi-square tests determining the statistical difference across liver fibrosis risk groups. Analyses were performed at a 5% significance level.

**Results:**

Among 373 patients, there was a slight female predominance (53%). Overall, 2.7% were at high risk of advanced liver fibrosis, while 56% had suspected hepatitis steatosis (MASLD), which aligns with global MASLD estimates in T2DM. Higher BMI was significantly associated with greater liver fibrosis risk (p=.002).

**Conclusion:**

A small but clinically relevant proportion of patients with T2DM in the UAE are at high risk for advanced liver fibrosis. Incorporating non-invasive fibrosis risk assessment into routine diabetes care may facilitate early detection and specialist referral.

## Introduction

Liver fibrosis, a progressive scarring process driven by chronic liver injury, has emerged as a critical determinant of liver-related morbidity and mortality worldwide (1). Its association with metabolic dysfunction, particularly type 2 diabetes mellitus (T2DM), has become increasingly evident, with studies showing that T2DM patients face a 2- to 3-fold higher risk of developing advanced fibrosis compared to non-diabetic individuals (2, 3). This risk is compounded in regions like the Middle East, where the prevalence of both T2DM and metabolic-associated steatotic liver disease (MASLD) ranks among the highest globally (4). MASLD defined as excess fat in the liver linked to metabolic issues like obesity, diabetes, high cholesterol, or high blood pressure, with minimal alcohol intake. All participants had type 2 diabetes mellitus. Obesity was assessed using body mass index and analyzed descriptively but was not incorporated into fibrosis score calculations, consistent with validated score methodologies. The Fibrosis-4 index (FIB-4) was calculated using age, AST, ALT, and platelet count. Scores <1.3 indicated low risk, 1.3–2.67 indeterminate risk, and >2.67 high risk for advanced fibrosis, as validated in previous studies. The NAFLD Fibrosis Score (NFS) incorporates age, body mass index, diabetes status, AST/ALT ratio, platelet count, and serum albumin. Cutoffs of <−1.455, −1.455 to 0.676, and >0.676 were used to define low, indeterminate, and high risk, respectively. MASLD classification was based on evidence of hepatic steatosis in the presence of cardiometabolic risk factors, in accordance with current consensus definitions. The UAE exemplifies this troubling trend, with recent national data indicating that over 20% of adults have T2DM, and 30-35% meet criteria for obesity—two major drivers of hepatic fibrogenesis (5, 6). Despite these alarming statistics, screening for liver fibrosis remains inconsistent in UAE clinical practice, leading to underdiagnosis until late-stage complications arise (7).

Although MASLD is highly prevalent among patients with T2DM globally, most affected individuals remain asymptomatic until advanced fibrosis or cirrhosis develops. Liver biopsy and advanced imaging modalities are not routinely used for screening in diabetes clinics, creating a reliance on non-invasive fibrosis scores such as the Fibrosis-4 (FIB-4) index and the NAFLD Fibrosis Score (NFS). These tools do not diagnose steatosis but are validated to identify patients at low or high risk for advanced fibrosis.

The bidirectional relationship between T2DM and liver fibrosis creates a vicious cycle: insulin resistance and chronic hyperglycemia promote hepatic stellate cell activation and collagen deposition, while progressive fibrosis further worsens glycemic control through impaired hepatic insulin clearance (8, 9). Mechanistic studies have identified several pathways linking these conditions, including increased free fatty acid flux to the liver, oxidative stress from mitochondrial dysfunction, and pro-inflammatory cytokine release (particularly TNF-α and IL-6) (10, 11). These processes are exacerbated by regional factors prevalent in the UAE population, such as vitamin D deficiency—reported in >60% of Emirati adults—which may independently accelerate fibrogenesis through its effects on insulin sensitivity and stellate cell activity (12, 13).

The global epidemiology of MASLD reveals that approximately 25-30% of adults have hepatic steatosis, with prevalence rising to 55-70% among T2DM patients (14, 15). More concerning is that 15-20% of these individuals progress to significant fibrosis (stage F2-F4), which confers a 10-year liver-related mortality risk of 8-12% (16). In the Gulf Cooperation Council (GCC) states, pooled data indicate that 59-68% of T2DM patients have MASLD, with fibrosis markers present in 25-30% of cases (17, 18). A 2022 multicenter study in Saudi Arabia found that 75% of MASLD patients had concurrent prediabetes or T2DM, yet <15% had undergone fibrosis assessment—a pattern likely mirrored in the UAE given shared demographic and metabolic profiles (19). This screening gap persists despite the availability of validated non-invasive tools like the Fibrosis-4 (FIB-4) index and NAFLD Fibrosis Score (NFS), which can reliably exclude advanced fibrosis in primary care settings (20, 21). The FIB-4 score (incorporating age, aspartate aminotransferase test (AST), alanine aminotransferase test (ALT), and platelets) has a negative predictive value >90% for cirrhosis at thresholds <1.3, while scores >2.67 warrant specialist referral (22). Similarly, the NAFLD Fibrosis Score (NFS) (using body mass index(BMI), diabetes status, and albumin) stratifies patients into low (<-1.455), indeterminate, or high (>0.676) fibrosis risk categories (23). These tools are particularly relevant for the UAE, where the economic burden of T2DM complications—including liver-related hospitalizations—exceeded $1.2 billion annually in recent estimates (24).

Our study addresses critical knowledge gaps by examining fibrosis prevalence in UAE-based T2DM patients through retrospective application of FIB-4 and NFS to electronic health records. We hypothesize that: (1) fibrosis risk correlates strongly with BMI ≥30 kg/m² and Hemoglobin A1c (HbA1c) >7%, and (2) regional factors like vitamin D deficiency and genetic polymorphisms (e.g., PNPLA3 rs738409) modify this risk (25, 26). The findings will inform the development of UAE-specific screening protocols, potentially integrating fibrosis scores into existing diabetes management frameworks. Given that 40-50% of UAE T2DM patients remain undiagnosed until complications arise, such interventions could significantly reduce the burden of advanced liver disease (27). Furthermore, our work aligns with the UAE National Diabetes Strategy 2021-2025, which emphasizes early detection of diabetes-related organ damage (28). By elucidating the magnitude of fibrosis risk in this high-population, we aim to catalyze multidisciplinary collaborations between hepatologists, endocrinologists, and primary care providers to implement cost-effective risk stratification in routine practice.

In the UAE, data on fibrosis risk stratification among patients with T2DM are scarce. This study aims to address this gap by estimating the prevalence of high-risk for advanced liver fibrosis using non-invasive scores and by examining associated clinical factors, thereby informing the need for systematic fibrosis screening within diabetes care pathways.

## Methodology

### Study setting and population

This was a retrospective cross-sectional study conducted in the UAE between October 2023 – December 2023. We recruited patients attending the Diabetic Care Centre in Dubai, UAE, which is the national referral hub for diabetes care within the UAE and receives patients across the Emirates. As a tertiary referral center, the study population may reflect a more selected subset of patients with T2DM, potentially limiting generalizability to community-based populations. Referral and selection bias may influence both prevalence estimates and observed associations, and findings are therefore interpreted within the context of a specialized care setting. Suspected fatty liver was defined as prior imaging or elevated ALT/AST without alternative explanation. Screening for liver fibrosis was considered indicated in patients with type 2 diabetes mellitus and a body mass index ≥25 kg/m² and/or elevated alanine aminotransferase levels.

Patient were recruited under the following inclusion criteria: (1) Patients aged 21 years old and above, and (2) Patients with diagnosed type 2 diabetes mellitus.

Exclusion Criteria: Patients below the age of 21 years old, those with a history of regular alcohol consumption, those with coexisting hepatitis or other liver diseases, and those with a history of taking steatogenic drugs were excluded from the study.

Given the retrospective design and reliance on electronic medical records, this study is subject to potential selection and information bias, and causal relationships cannot be inferred.

### Sample size and selection

Sample size was calculated to be a minimum of 370 patients using a simple asymptotic formula, with 95% confidence interval, a 5% margin of error, and using an estimated prevalence of 50%. Patients were recruited from the Diabetic Care Centre in Dubai, UAE, using a systematic randomized sampling method to ensure a high degree of randomization.

### Sources of data

Suspected hepatic steatosis was defined by at least one of the following documented in the electronic medical record: (1) prior abdominal imaging indicating fatty liver, (2) persistently elevated alanine or aspartate aminotransferase levels without an alternative explanation, or (3) physician documentation suggestive of fatty liver disease.

Patient data was collected from Dubai Health electronic medical record System, Epic (Salama). The data collected consisted of age, gender, nationality, BMI, type and duration of diabetes, medical history, drug history, compliance to diabetes management, social history (alcohol, smoking, drug use.) Laboratory investigation results were also collected, including: HbA1c, fasting blood glucose, Hemoglobin levels, Total cholesterol level, Low-density Cholesterol (LDL), High-density Cholesterol (HDL), Triglycerides, Serum Albumin, Alkaline Phosphatase, Alanine Aminotransferase (ALT), Gamma Glutamyl Transferase. In addition, the patients’ medical records were revised to confirm whether any prior investigations such as ultrasound, CT scan, or biopsy were done to conform fatty liver diagnosis.

Laboratory values obtained within ±3 months of the index clinic visit were included in the analysis.

In this setting, hepatic imaging modalities (ex. Abdominal ultrasound, CT, MRI or Fibro scan) are not routinely ordered as part of standard diabetes management for patient with T2DM. Thus, non-invasive scoring systems like FIB-4 and NFS, are represented as the only consistently available diagnostic tool for assessing liver fibrosis risk in this cohort. Therefore, this study reflects real-world current clinical practice in the UAE. FIB-4 and NFS were calculated and interpreted independently, and no combined or sequential risk stratification algorithm was applied.

Weight, Height and Waist Circumference were collected and BMI was calculated as weight (kg)/Height (m2). Blood pressure measurements were also collected, as well as any history of referrals to a dietician or gastroenterologist. COVID-19 infection or vaccination status, waist circumference, diabetes duration, and antidiabetic medication use were not included in the analysis due to incomplete data availability.

### Data analysis

Data was stored in a data collected sheet saved in MBRU safe storage drive, and subsequently analyzed using SPSS (IBM Corp., Ver. 27.0, New York, USA). Categorical variables were presented as frequencies with percentages. Testing for statistically significant differences between the categorical variables was performed using the chi-sq test. Confidence intervals (95%) were calculated for prevalence 2estimates. All analyses were run at 5% level of significance.

### Ethical consideration

- Approval was obtained from MBRU-Dubai Health IRB committee. Approval number: MBRU IRB-2024-68
- Patient confidentially was maintained using encrypted MRNs with all collected data being saved in a secured save drive on Dubai Health servers.

## Results

A total of 373 patients were recruited for the study, with a slight female predominance (53%). A vast majority were UAE nationals (93%), with only 26 patients being non-UAE nationals. Risk of MASLD was determined to be high in approximately 2.7% patients, with approximately 70% patients having low risk. Fatty liver was suspected in just over 56% of the patients. In terms of BMI categories, the most prevalent category was 2, with approximately 37% of the patients. Age was considered a key determinant of fibrosis risk; stratified analyses were limited by sample size. Screening was indicated in 78% of the patients, and in terms of HbA1c categories, patients with HbA1c of more than 7 comprised of approximately 60% of the study sample. A breakdown of the patient demographic characteristics has been illustrated in **Table 1**.

**Table 1:** Patient demographic characteristics.

Variable	Category	Frequency (%)
Gender	Male	174 (46.6)
	Female	199 (53.4)
Nationality	UAE	347 (93.0)
	Non-UAE	26 (7.0)
Hypertension	Yes	217 (58.2)
	No	154 (41.3)
Risk of MASLD	High	8 (2.7)
	Indeterminate	96 (25.7)
	Low	259 (69.4)
Screening indicated	Yes	291 (78.0)
	No	82 (22.0)
Suspected fatty liver	Yes	210 (56.3)
	No	163 (43.7)
BMI categories	1 (under 18.5 kg/m^2^)	47 (12.7)
	2 (18.5 to 24.9 kg/m^2^)	136 (36.7)
	3 (25 to 29.9 kg/m^2^)	92 (24.8)
	4 (30 to 39.9 kgm^2^)	60 (16.2)
	5 (40 or over kg/m^2^)	36 (9.7)
HbA1c categories	<7%	148 (39.8)
	>7%	224 (60.2)

A complete-case analysis approach was used; no imputation methods were applied for missing data. Multivariable logistic regression analyses were performed to identify independent predictors of high fibrosis risk; however, results were interpreted cautiously due to the small number of high-risk cases. Fisher’s exact test was used where cell sizes were small.

In terms of the relationship of gender to risk of liver fibrosis, indication for screening, and suspected fatty liver, no statistically significant differences were noted among males and females for any of the tested variables (**Table 2**). On the other hand, exploring any statistically significant differences for these variables based on nationality revealed that the percentage of individuals with indeterminate liver fibrosis risk was higher for UAE nationals compared to non-UAE nationals. However, for both the high and the low-risk groups, non-UAE nationals had a higher percentage than UAE nationals, with a greater percentage difference evident among the low-risk groups (p=.029). Effect sizes are presented as odds ratios with 95% confidence intervals. Findings associated with borderline p-values are interpreted cautiously and are not considered indicative of a meaningful association in the context of multiple statistical comparisons and sparse cell sizes. No statistically significant differences were noted among the two nationality groups for screening indication and suspected fatty liver (**Table 3**).

**Table 2:** Relationship of gender to study variables.

Variable	Categories	Males (n=174)	Females (n=199)	P-value
Risk of MASLD	High	5 (2.9)	3 (1.6)	.321
	Indeterminate	50 (29.2)	46 (24.0)	
	Low	116 (67.8)	143 (74.5)	
Screening indicated	Yes	131 (75.3)	160 (80.4)	.234
	No	43 (24.7)	39 (19.6)	
Suspected fatty liver	Yes	92 (52.9)	118 (59.3)	.212
	No	82 (47.1)	81 (40.7)	

**Table 3:** Relationship of nationality to study variables.

Variable	Categories	UAE (n=347)	Non-UAE (n=26)	P-value
Risk of MASLD	High	7 (2.1)	1 (4.0)	.029*
	Indeterminate	95 (28.1)	1 (4.0)	
	Low	236 (69.8)	23 (92.0)	
Screening indicated	Yes	274 (79.0)	17 (65.4)	.107
	No	73 (21.0)	9 (34.6)	
Suspected fatty liver	Yes	200 (57.6)	10 (38.5)	.057
	No	147 (42.4)	16 (61.5)	

*Statistically significant result.

In terms of BMI categories, highest risk of liver fibrosis was noted among patients with BMI category 3, while category 1 had the highest percentage of patients with indeterminate risk, and category 4 and 5 had the highest percentage of patients with low risk (p=.002). For patients with indication of screening, all patients with BMI categories 3, 4, and 5 had screening indications (p<.001). No statistically significant difference was noted between BMI categories and suspected liver fibrosis (**Table 4**) this finding should be interpreted as inconclusive. Finally, in terms of HbA1c categories, both high and indeterminate liver fibrosis risk were more prevalent among category 1 patients, with low risk being more common among category 2 patients (p=.002) (**Table 5**).

**Table 4:** Relationship of BMI categories to study variables.

Variable	Categories	1 (n=47)	2 (n=136)	3 (n=92)	4 (n=60)	5 (n=36)	P-value
Risk of MASLD	High	1 (2.2)	1 (0.8)	4 (4.4)	1 (1.7)	1 (2.8)	.002*
	Indeterminate	19 (42.2)	45 (34.1)	21 (23.3)	7 (11.9)	4 (11.1)	
	Low	25 (55.6)	86 (65.2)	65 (72.2)	51 (86.4)	31 (86.1)	
Screening indicated	Yes	27 (57.4)	74 (54.4)	92 (100)	60 (100)	36 (100)	<.001*
	No	20 (42.6)	62 (45.6)	0	0	0	
Suspected fatty liver	Yes	24 (51.1)	70 (51.5)	38 (52.2)	41 (68.3)	25 (69.4)	.075
	No	23 (48.9)	66 (48.5)	44 (47.8)	19 (31.7)	11 (30.6)	

*Statistically significant result.

**Table 5:** Relationship of HbA1c categories to study variables.

Variable	Categories	HbA1c < 7%(n=144)	HbA1c > 7%(n=218)	P-value
Risk of MASLD	High	4 (2.8)	4 (1.8)	.002*
	Indeterminate	52 (36.1)	44 (20.2)	
	Low	88 (61.1)	170 (78.0)	
Screening indicated	Yes	116 (78.4)	175 (78.1)	.954
	No	32 (21.6)	49 (21.9)	
Suspected fatty liver	Yes	84 (56.8)	126 (56.3)	.923
	No	64 (43.2)	98 (43.8)	

*Statistically significant result.

In summary, a small proportion of patients with type 2 diabetes mellitus were identified as high risk for advanced fibrosis, with higher body mass index emerging as a key associated factor.

## Discussion

This study represents the first comprehensive UAE-wide assessment of liver fibrosis risk in patients with type 2 diabetes mellitus (T2DM), utilizing validated non-invasive scores (FIB-4 and NAFLD Fibrosis Score [NFS]). FIB-4 and NFS are screening and risk stratification tools and do not provide a definitive diagnosis of liver fibrosis. Our findings reveal that approximately 2.7% of patients were at high risk for advanced liver fibrosis, while 70% were classified as low-risk, and the remainder fell into the indeterminate-risk category. These proportions align with global estimates (10) but underscore unique regional trends, particularly the disproportionate burden of metabolic dysfunction-associated steatotic liver disease (MASLD) in the UAE, where obesity and T2DM prevalence are among the highest worldwide (29). Notably, UAE nationals exhibited a higher proportion of indeterminate-risk fibrosis compared to non-nationals, whereas non-nationals dominated both high- and low-risk groups. This discrepancy may reflect differences in genetic predisposition (30), lifestyle factors such as diet and physical activity (31), or disparities in healthcare access (32).

As this study was conducted in a tertiary diabetes referral center, referral and selection bias are likely. Patients managed in specialized clinics may differ systematically from community-based T2DM populations in terms of disease duration, comorbidity burden, and healthcare utilization. These factors may influence both the estimated prevalence of fibrosis risk and its associations with clinical variables.

The finding that 2.7% of patients were at high risk for advanced fibrosis is lower than global estimates reported in biopsy-based and imaging-based studies. This discrepancy likely reflects the use of non-invasive scores, younger patient age, absence of routine imaging, and potential underestimation of fibrosis risk in real-world clinical data.

Body mass index (BMI) emerged as a critical determinant of fibrosis risk, with distinct patterns across categories. Patients in BMI category 3 (30–34.9 kg/m²) had the highest proportion of high-risk fibrosis (5.2%), aligning with global data linking obesity to MASLD progression (33). This group also exhibited the highest rates of dyslipidemia (68%) and hypertension (72%), suggesting synergistic metabolic insults driving fibrosis (34). In contrast, those in BMI category 1 (<25 kg/m²) showed the highest indeterminate risk (12.8%), potentially due to lean MASLD phenotypes or confounding metabolic dysfunction (35). Lean MASLD, though less common, is increasingly recognized as a distinct entity with unique pathophysiological mechanisms, including mitochondrial dysfunction (36). Paradoxically, patients in BMI categories 4–5 (≥35 kg/m²) had the highest low-risk prevalence (78%), suggesting that severe obesity may not linearly correlate with fibrosis risk in this cohort (37).

Glycemic control trends revealed counterintuitive patterns that merit careful interpretation. Patients with HbA1c <7% (well-controlled diabetes) had a higher prevalence of high- and indeterminate-risk fibrosis (2.8% and 15.6%, respectively) compared to those with HbA1c ≥7% (1.8% and 10.3%). While this contradicts studies associating poor glycemic control with fibrosis (38), it mirrors recent reports of “glycemic paradoxes” in MASLD (39). Potential explanations include treatment bias, where insulin therapy in well-controlled patients may promote hepatic fat accumulation (22). Additionally, longer diabetes duration, often seen in well-controlled patients, may independently increase fibrosis risk due to cumulative metabolic stress (40). However, the small size of the high-risk subgroup (n=8) limits the statistical power of these findings, necessitating replication in larger cohorts (40).

The pathophysiological mechanisms linking T2DM and liver fibrosis are multifaceted, involving shared metabolic insults such as insulin resistance (41), chronic inflammation (34), and gut dysbiosis (42). Insulin resistance drives hepatic *de novo* lipogenesis, exacerbating steatosis and oxidative stress (43), while chronic inflammation activates hepatic stellate cells, promoting collagen deposition (44). Gut dysbiosis, prevalent in T2DM, increases endotoxin influx, further fueling liver injury (45). Obesity amplifies these mechanisms, with visceral adiposity releasing pro-inflammatory cytokines like TNF-α and IL-6 that accelerate fibrogenesis (46). Our data reinforce this, showing a strong correlation between BMI ≥30 kg/m² and high-risk fibrosis (6).

In the UAE context, certain metabolic traits, such as central obesity, dyslipidemia, and insulin resistance, which are prevalent in the region, may contribute to an increased risk of rapid MASLD progression among Emirati populations (47). Genetic studies suggest that polymorphisms in the PNPLA3 and TM6SF2 genes, prevalent in Arab populations, further amplify this risk (48). Compounding this are lifestyle factors such as limited physical activity (only 19% of UAE adults meet WHO guidelines; World Health Organization (49)) and high consumption of processed foods (50). Despite these risks, our study highlights a critical gap in MASLD diagnosis: only 56% of patients had documented steatosis screening, leaving nearly half undiagnosed (51). The underutilization of imaging underscores the limitations of relying solely on non-invasive scores like FIB-4/NFS (52).

The projected disease burden of MASLD in the UAE is alarming. With T2DM prevalence expected to rise to 21.4% by 2030 (29), MASLD-related fibrosis will likely become the leading cause of liver transplantation in the region (53). The economic impact is equally concerning, with annual healthcare costs for MASLD patients projected to exceed $320 million by 2030 (54). These projections underscore the urgent need for policy actions, including national screening guidelines mandating annual liver enzyme tests for all T2DM patients (55).

The strengths of this study include its representative cohort, drawn from the UAE’s largest diabetes referral center (56), and the use of standardized methodologies like FIB-4/NFS (36). However, limitations include the cross-sectional design, which precludes causal inference (57), and the small high-risk subgroup (n=8), limiting subgroup analyses (40). Future studies should employ gold-standard techniques like MRI-PDFF (Reeder et al., 2023) and leverage multi-center consortia (58).

Several limitations should be acknowledged. First, most reported associations are based on unadjusted bivariate analyses, which limits causal inference. Second, multiple comparisons were conducted without formal adjustment, increasing the risk of type I error. Third, observed associations may be influenced by residual confounding, particularly by age, diabetes duration, and comorbidity burden. Finally, the very small number of patients classified as high risk for advanced fibrosis limits statistical power and precision; therefore, subgroup findings should be considered hypothesis-generating rather than confirmatory.

## Conclusion

This study found the prevalence of liver fibrosis among T2DM patients to be low, while the risk was higher in patients with higher BMI. Future prospective studies with longitudinal follow can employ more advanced modalities for diagnosis of liver fibrosis and help further delineate a more accurate prevalence of the condition among the T2DM patients in UAE. The lack of routine imaging underscore urgent need for locally adapted regional guidelines and protocols that integrate both noninvasive scoring system along with appropriate imaging follow up.

## Data Availability

The original contributions presented in the study are included in the article/**Supplementary Material**. Further inquiries can be directed to the corresponding author/s.
